# Understanding obesity in children with 22q11.2 deletion syndrome

**DOI:** 10.3389/fendo.2025.1628796

**Published:** 2025-07-31

**Authors:** Walter Maria Sarli, Matteo Cerutti, Matteo Pontone, Valentina Guarnieri, Sara Soldovieri, Massimo Giotta, Silvia Ricci, Chiara Azzari, Stefano Stagi

**Affiliations:** 1 Immunology Unit, Meyer Children’s Hospital IRCCS, Florence, Italy; 2 Department of Health Sciences, University of Florence, Florence, Italy; 3 PhD School in Public Health, Clinical Medicine and Oncology, Department of Precision and Regenerative Medicine and Ionian Area, School of Medicine, University of Bari Aldo Moro, Bari, Italy; 4 Diabetology and Endocrinology Unit, Meyer Children's Hospital IRCCS, Florence, Italy

**Keywords:** 22q11 deletion syndrome, DiGeorge, obesity, overweight, thyroid, neuropsychiatric disease, prevention, children

## Abstract

**Background:**

22q11.2 deletion syndrome (22q11.2DS) is a complex and heterogeneous genetic disorder. While short stature is well-documented, data on weight excess in pediatric patients remain sparse and conflicting.

**Objective:**

To evaluate the prevalence of overweight and obesity in children and adolescents with 22q11.2DS when compared to the general Italian pediatric population and identify risk factors and periods of critical weight gain.

**Methods:**

In this single-center, retrospective study, 61 children with molecularly confirmed 22q11.2DS were followed up for 441 patient-years. Anthropometric data were evaluated and compared to national surveillance databases (“OKKio alla Salute” and “HBSC”). Risk factors for overweight and obesity were identified by univariate and multivariate analyses.

**Results:**

While overweight prevalence in 22q11.2DS patients did not differ significantly from that in the general population, obesity had a bimodal age distribution with peaks at 11 and 17 years. Both neuropsychiatric comorbidities and the use of psychoactive medication were significantly associated with an increased risk of overweight and obesity. In multivariate analysis, the use of psychoactive medication was the only independent risk factor.

**Conclusions:**

Obesity in 22q11.2DS may not be syndrome-intrinsic but is heavily influenced by pharmacological treatment. Identification of vulnerable periods and modifiable risk factors is crucial. A preventive, multidisciplinary approach with metabolic screening and cautious use of psychotropic medication is warranted to avoid obesity risk in this population.

## Introduction

22q11.2 deletion syndrome (22q11.2DS) (OMIM #188400/#192430) is the most common chromosomal microdeletion disorder (1). It results from *de novo* non-homologous meiotic recombination events in more than 90% of cases (2) and displays high penetrance. The prevalence has been estimated to range from 1 per 3,000 to 1 per 6,000 live births, basing the diagnosis on major birth defects or on a few population screening studies (3–6). However, a recent analysis of 30,000 samples of neonatal dried blood spots estimated a minimum prevalence of 1 in 2,148 live births (7), while other studies evidence that the deletion occurs in approximately 1 in every 1,000 fetuses (8, 9).

Most of the phenotypic features associated with 22q11.2DS are due to altered morphogenesis of the pharyngeal arch system, particularly from the third and fourth branchial arches. All tissues originating from the branchial arches system can be affected: craniofacial structures, thymus, parathyroid glands, aortic arch and cardiac outflow tract are the main involved (1). Nevertheless, the numerous amounts of genes settled in this chromosomal region combined with the natural predisposition to non-homologous recombination (10), makes the phenotype extremely heterogenous, irrespectively of deletion size (1, 11).

Moreover, the phenotypical features that lead to diagnosis change during patient’s life: congenital heart defects, hypocalcemia, chronic infections, feeding difficulties, developmental and language delays, behavioral and learning disabilities (12–15) are typical of newborns and infants, while adolescents and adults can manifest behavioral abnormalities, learning difficulties and even psychiatric illness such as anxiety disorders and schizophrenia (2, 15, 16).

From an auxological point of view, short stature is reported to be a relatively frequent finding (17–19). Poor growth may be the result of intrauterine growth retardation (IUGR), cardiopathy, velopharyngeal abnormality and feeding difficulties, recurrent infections and, in few cases, growth hormone deficiency (GHD) (17). IGF-1 levels can be reduced by poor nutrition, severe hepatic disease, poorly controlled diabetes mellitus, and inadequately treated hypothyroidism (20). Late adolescent and adult’s stature is usually in the normal range according to several authors (11, 13, 21) while other studies highlights that a substantial percentage of adult individuals remain below the third percentile (22, 23).

The prevalence of obesity (1, 22, 24), type 2 diabetes mellitus (24, 25) and hypertriglyceridemia (26) is significantly higher among adults compared to the general population”. To date, only limited pediatric data are available, and the results reported in the literature have been inconsistent.

The aim of the study was to describe overweight and obesity in a pediatric population of children affected by 22q11.22DS and to compare it to general Italian pediatric population. We also aimed to understand if weight gain occurs at a certain age stage. Lastly, we aimed to identify predisposing conditions for weight gain to deploy the wisest preventive strategy in the real-life setting.

## Materials and methods

### Study design

This single-center, longitudinal and retrospective study aimed to evaluate the prevalence of weight gain in pediatric patients with 22q11.2DS compared with that of the general Italian pediatric population, using data from two surveillance programs: “OKKio alla Salute” and “Health Behaviour in School-aged Children” (HBSC). “OKKio alla Salute” is a national nutritional surveillance program, part of the Childhood Obesity Surveillance Initiative (COSI) of the World Health Organization (WHO) Regional Office for Europe, established with the aim of monitoring the nutritional status and lifestyle behaviors among children in the third grade of primary schools, aged 8–9 years (27). HBSC is an international multicenter study conducted in more than 40 countries across Europe and North America, in collaboration with the WHO Regional Office for Europe, with the aim of monitoring the nutritional status in school young adolescents aged 11-13-15–17 years (28).

A secondary aim of the study was to investigate potential clinical and demographic risk factors for early-onset weight gain, in order to identify patients with 22q11DS who are at high risk of overweight or obesity in childhood or adolescence.

### Inclusion criteria and clinical definitions

Patients with a confirmed molecular diagnosis of 22q11.2 deletion syndrome (22q11DS), diagnosed before 18 years of age, were considered eligible for inclusion. All patients were followed at the Pediatric Endocrinology and Immunology Divisions of Meyer Children’s Hospital in Florence, Italy.

Eligible patients were those who attended follow-up visits between April 1st, 1985, and August 31st, 2024, and were monitored at least annually until the age of 18.

Clinical data were retrospectively collected from medical records, using a standardized and anonymized data collection form.

Anthropometric assessments followed international standards.

Stature was measured as standing height (SH) in children older than 2 years using a Harpenden-Holtain stadiometer, or as supine length (SL) in those younger than 2 years using a rigid measuring board. Height, or length, was expressed in centimeters and converted to standard deviation scores (SDS) according to Italian reference growth charts (29).

Body weight was measured with a calibrated digital scale and expressed in kilograms (Kg).

Nutritional status was assessed using weight-for-length (WFL) in children under 2 years, and body mass index (BMI), calculated as weight in kilograms divided by height in meters squared, in children aged 2–18 years.

Overweight was defined as WFL ≥ +2 SDS or BMI ≥ +1 SDS, and obesity as WFL ≥ +3 SDS or BMI ≥ +2 SDS.

Neonatal auxological data were also collected. Small-for-gestational-age (SGA) status was defined as a birth weight and/or length below -2 SDS for gestational age, according to the latest consensus criteria (30).

To enable a fair comparison with general pediatric population data from national surveillance programs, growth and nutritional outcomes were stratified into five discrete age categories: 8.00–8.99, 11.00–11.99, 13.00–13.99, 15.00–15.99, and 17.00–17.99 years.

Additionally, selected comorbidities potentially affecting weight and growth were documented, including thyroid dysfunction, congenital heart disease, psychiatric disorders, and the use of psychotropic medications.

### Statistical analysis

Quantitative data were expressed as mean and standard deviation (SD) if normally distributed, and as median and interquartile range (IQR) if the assumption of normality was not acceptable. The Shapiro-Wilk statistic was used to test for normality. Differences in continuous variables between groups were compared using the Student’s t-test for normally distributed parameters, or the nonparametric Mann-Whitney U test otherwise. Categorical data were expressed as frequency and percentage, and the Chi-square test or Fisher’s exact test were used to compare groups. Yates’ correction for continuity was applied depending on the number of observations. Univariate and multivariate Cox regression models were applied to assess the effect of variables (sex, presence of heart disease, presence of thyroiditis, use psychoactive drug and neuropsychiatric disorder) on risk of overweight and obesity. Using the p-values criterion (p < 0.25), a stepwise selection was used to estimate the final model. Cox model results are shown using adjusted hazard ratios (aHR) with their 95% confidence interval (CI).

All tests of statistical significance were two-tailed, and *p*-values less than 0.05 were considered statistically significant. Statistical analysis was performed using the SAS/STAT^®^ Statistics, Version 9.4 (SAS Institute Inc., Cary, NC, USA).

## Results

A total of 61 patients (26 females, 35 males) met the inclusion criteria. The study is based on a long follow-up period (441 patient-years). The mean duration of follow-up for enrolled patients was 7.22 ± 3.48 years, the minimum follow-up period was 1 year, and the maximum follow-up period was 13 years.

The cumulative incidence of overweight across all ages was 27.87%, with a median age of onset of 9.76 years (IQR 7.02), while the cumulative incidence of obesity was 24.59%, with a median age of onset of 5.31 years (IQR 7.97), as reported in **Table 1A**. Stratification by sex revealed no significant differences between males and females for either overweight (58.82% overweighed males *versus* 56.82% non-overweighed males, *p=1.00*) or obesity (66.67% obese males *versus* 54.35% non-obese males, *p=.55*).

Table 1Prevalence, cumulative incidence, and age-related trends in overweight and obesity among patients with 22q11.2 deletion syndrome, with comparison to national surveillance data.

**Table:** 

A				
Condition	Patients affected (n)	Cumulative incidence (%)	Median age of onset (years)	IQR (years)
**Overweight**	17	27.87%	9.76	7.02
**Obesity**	15	24.59%	5.31	7.97

**Table:** 

B							
Age group	Patients (n)	Overweight			Obesity		
		Prevalence (%)	Male/female (n/n)	BMI (Mean ± SD)	Prevalence (%)	Male/female (n/n)	BMI (Mean ± SD)
**8 years**	24	12.5	1/2	19.35 ± 1.95	16.7	3/1	23.70 ± 2.96
**11 years**	25	16	1/3	21.59 ± 1.59	16	4/0	26.42 ± 2.11
**13 years**	27	11.11	1/2	25.29 ± 1.03	11.11	2/1	30.29 ± 1.92
**15 years**	23	17.39	1/3	25.95 ± 1.79	8.69	2/0	31.66 ± 1.13
**17 years**	19	21.05	2/2	26.13 ± 1.88	26.31	3/2	33.31 ± 3.84

**Table:** 

C															
Outcome measure	8 years			11 years			13 years			15 years			17 years		
	Our cohort	OKKIO alla salute	*p-value*	Our cohort	HSBC	*p-value*	Our cohort	HSBC	*p-value*	Our cohort	HSBC	*p-value*	Our cohort	HSBC	*p-value*
**Patients (n)**	**24**	46559		**25**	21489		**27**	23077		**23**	22187		**19**	22568	
**Overweight (prevalence, %)**	**12,5**	19	**.54**	**16**	19.3	**.70**	**11,11**	18.3	**.38**	**17,39**	17	**.96**	**21,05**	15.9	**.57**
**Obesity (prevalence, %)**	**16,7**	9.8	**.32**	**16**	5.0	**.01**	**11.11**	4.3	**.09**	**8,69**	3.9	**.24**	**26,31**	3.4	**<.001**

**A.** The cumulative incidence of overweight and obesity, with corresponding median age of onset and interquartile range (IQR). Age of onset was defined as the first chronological time point in which the patient met the BMI criteria for overweight (≥ +1 SDS) or obesity (≥ +2 SDS). **B.** Prevalence of overweight and obesity, along with the mean and standard deviation (SD) of Body Mass Index (BMI), across different age groups (8, 11, 13, 15, and 17 years) in our cohort. Male/female ratio is presented. The prevalence of obesity exhibits a bimodal distribution, with peaks at 11 years and 17 years (33.31%), and a decrease at 15 years. **C.** Prevalence of overweight and obesity in our cohort of patient with 22q11.2 DS and comparison with Italian pediatric population data (Italian Surveillance Systems). The prevalence of obesity shows a bimodal increase when compared to the general pediatric population, with statistically significant results observed in the 11-year-old and 17-year-old age groups. Legend: n, number. Bold values indicate statistically significant results (p < 0.05).

The differences in BMI in relation to age and gender are presented in **Table 1B**. The prevalence of obesity exhibited a bimodal distribution, with peaks at 11 years (16%) and 17 years (26.31%), and a notable decrease at 15 years (8.69%). Pairwise comparisons between age groups did not reveal any statistically significant differences. Due to the limited sample size, stratification by sex within individual age groups was not feasible.

When comparing the prevalence of overweight in our cohort with that of the general population, as per current screening programs, no significant differences were found. In contrast, the prevalence of obesity showed a bimodal increase relative to the general population, with statistically significant results observed in the 11-year-old (*p=.01*) and 17-year-old (*p<.001*) age groups (**Table 1C**).

**Figure 1:**
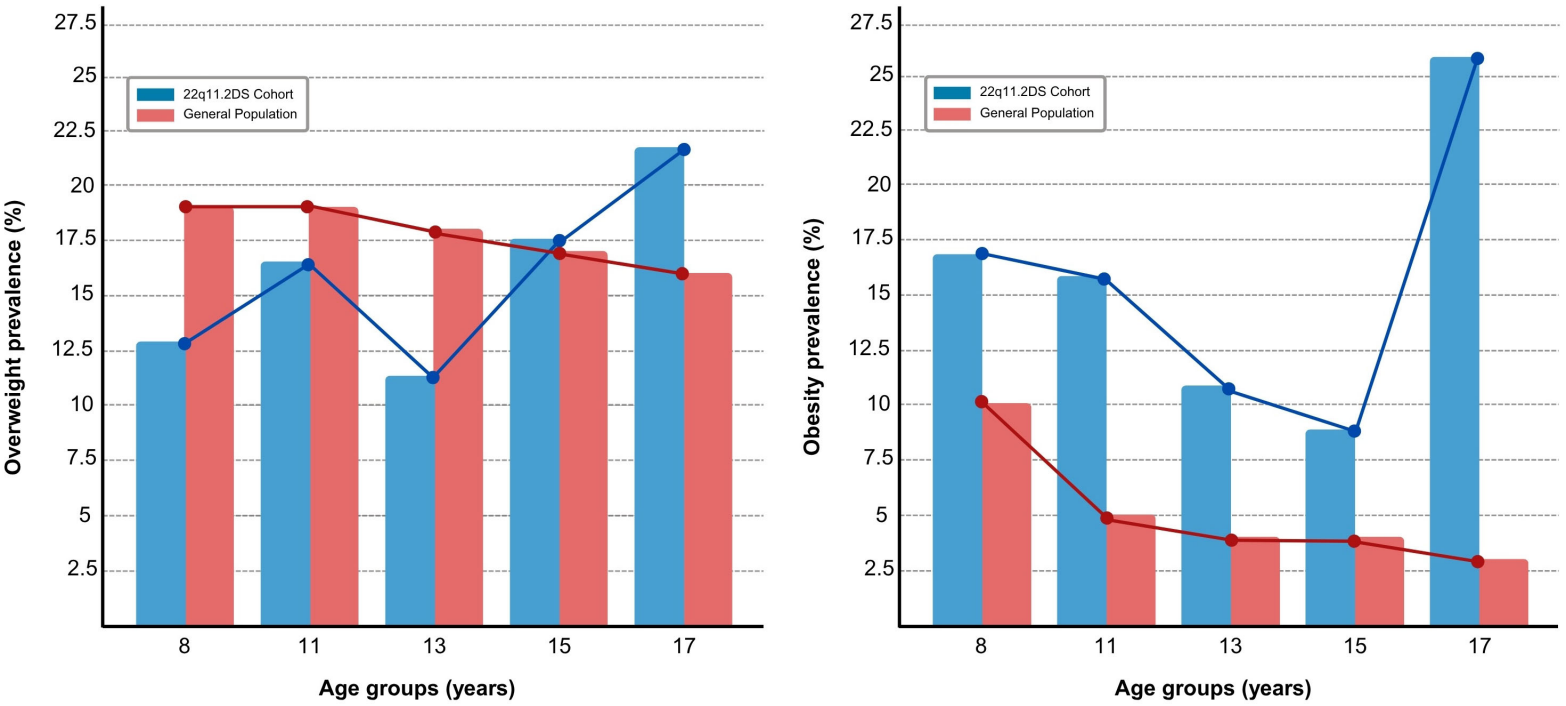
Prevalence of overweight (left panel) and obesity (right panel) across age groups (8, 11, 13, 15, 17 years) in the 22q11.2DS cohort (blue bars) compared to the general population (red bars). The line plots represent the trends in prevalence for each group.

The trend in the prevalence of obesity and overweight across different age groups is presented in **Figure 1**.

The prevalence of hypothesized risk factors, such as being SGA for weight, SGA for length, thyroid disease, heart disease, neuropsychiatric disorders, and use of psychoactive medications, among obese and non-obese patients, as well as between overweight and non-overweight patients, is shown in **Table 2**.

**Table 2:** Association between overweight and obesity and selected clinical and demographic factors in patients with 22q11.2 deletion syndrome.

Risk factors	Overweight YES	Overweight NO	p-value	Obese YES	Obese NO	*p*-value
SEX						
**Male**	**10 (58.8)**	**25 (56.82)**	**1**	**10 (66.67)**	**25 (54.35)**	**0.55**
**Female**	**7 (41.18)**	**19 (43.18)**		**5(33.33)**	**21 (45.65)**	
HEART DISEASE						
**Yes**	**7 (41.18)**	**29(65.91)**	**.09**	**9 (60)**	**27 (58.7)**	**1**
**No**	**10 (58.82)**	**15 (34.09)**		**6 (40)**	**19 (41.3)**	
THYROID DISEASE						
**Yes**	**5 (29.41)**	**9 (20.45)**	**.51**	**3 (20)**	**11 (23.91)**	**1**
**No**	**12 (70.59)**	**35 (79.55)**		**12 (80)**	**35 (76.09)**	
NEUROPSYCHIATRIC DISORDERS						
**Yes**	**8 (47.06)**	**7(15.91)**	**.02**	**9 (60)**	**6 (13.04)**	**.001**
**No**	**9(52.94)**	**37 (84.09)**		**6 (40)**	**40 (86.96)**	
PSYCHOACTIVE DRUG USE						
**Yes**	**6 (35.29)**	**3(6.82)**	**.01**	**7 (46.67)**	**2 (4.35)**	**<.001**
**No**	**11 (64.71)**	**41(93.18)**		**8 (53.33)**	**44 (95.65)**	
SGA FOR WEIGHT						
**Yes**	**0 (0)**	**7 (13.73)**	**1**	**1 (7.69)**	**14 (32.56)**	**.15**
**No**	**3 (100)**	**44 (86.27)**		**12 (92.31)**	**29 (67.44)**	
SGA FOR LENGHT						
**Yes**	**0 (0)**	**3 (7.69)**	**.55**	**0 (0)**	**3 (7.32)**	**1**
**No**	**15 (100)**	**36 (92.31)**		**13 (100)**	**38 (92.68)**	

Distribution of overweight and obesity status across various factors, including sex, heart disease, thyroid disease, neuropsychiatric disorders, psychoactive drug use, and small for gestational age (SGA) for weight and length. The p-values reflect statistical significance for each comparison.

No statistically significant differences were observed in the prevalence of thyroid disease (*p=.51* for overweight; *p=1.00* for obesity), heart disease (*p=.09* for overweight; *p=1.00* for obesity), SGA for weight (*p=1.00* for overweight; *p=.15* for obesity), or SGA for length (*p=.55* for overweight; *p=1.00* for obesity) between overweight and non-overweight patients, as well as between obese and non-obese patients. Conversely, both overweight and obese patients exhibited a significantly higher prevalence of neuropsychiatric comorbidities (*p=.02* for overweight; *p=.001* for obesity) and psychoactive drug consumption (*p=.01* for overweight; *p<0.001* for obesity) compared to their non-overweight and non-obese counterparts.

In details, 21.3% of our patients had at least one neuropsychiatric disorder and, among them, 16.4% had two or more disorders. Similarly, 18.0% received at least one psychoactive medication and 13.1% received two or more of them. Neuropsychiatric disorders and psychoactive medications of our cohort are listed in detail in **Tables 3**, **4**.

**Table 3:** Prevalence and classification of neuropsychiatric disorders in the study cohort.

A.			
Number of diagnosed neuropsychiatric disorders per patient	n (%)	Cumulative n (%)	
1	4 (6.6)	13 (21.3)	
2	6 (9.8)	10 (16.4)	
3	3 (4.9)	3 (4.9)	
B			
Neuropsychiatric categories according to DSM V	n/total cohort (%)	Specific disorder	n/total cohort (%)
**Neurodevelopmental disorders**	9/61 (14.8)	ADHD	5/61 (8.2)
		Intellectual disability	3/61 (4.9)
		Autism spectrum disorder	1/61 (1.6)
**Anxiety disorders**	5/61 (8.2)	Generalized anxiety	5/61 (8.2)
**Depressive disorders**	4/61 (6.6)	Disruptive mood dysregulation disorder	2/61 (3.3)
		Major depressive disorder	1/61 (1.6)
		Mixed disorder	1/61 (1.6)
**Disruptive, Impulse-Control and Conduct Disorder**	4/61 (6.6)	Conduct disorder	4/61 (6.6)
**Somatic Symptoms and related disorder**	1/61 (1.6)	Somatic symptoms	1/61 (1.6)
**Schizophrenia spectrum and related disorders**	1/61 (1.6)	Brief psychotic disorder	1/61 (1.6)
**Trauma and stressor related disorders**	1/61 (1.6)	Adjustment disorder	1/61 (1.6)

**A.** Number of neuropsychiatric disorders diagnosed per patient. The table shows the distribution and cumulative frequency of patients according to the number of distinct neuropsychiatric diagnoses received. **B.** Prevalence and distribution of neuropsychiatric disorders in the study cohort, including diagnostic categories and specific conditions according to DSM V. Percentages refer to the proportion of patients affected among the total study cohort (61 patients).

**Table 4:** Use and distribution of psychoactive medications in the study cohort.

A					
Number of psychoactive medications per patient		n (%)		Cumulative n (%)	
1		5 (8.2)		11 (18.0)	
2		3 (4.9)		8 (13.1)	
3		3 (4.9)		3 (4.9)	
B					
Pharmacological classes	n/total cohort (%)	n/patient with ≥1 psychoactive medication (%)	Medications	n/total cohort (%)	n/patient with ≥1 psychoactive medication (%)
Atypical antipsychotics	7/61 (11.5)	7/11 (35)	Risperidone	3/61 (4.9)	3/11 (27.3)
			Aripiprazole	2/61 (3.3)	2/11 (18.2)
			Quetiapine	1/61 (1.6)	1/11 (9.1)
			Clozapine	1/61 (1.6)	1/11 (9.1)
Mood stabilizers	6/61 (9.8)	6/11 (30)	Valproate	2/61 (3.3)	2/11 (18.2)
			Carbamazepine	1/61 (1.6)	1/11 (9.1)
			Lamotrigine	1/61 (1.6)	1/11 (9.1)
			Topiramate	1/61 (1.6)	1/11 (9.1)
			Lithium	1/61 (1.6)	1/11 (9.1)
Antidepressants	3/61 (4.9)	3/11 (15)	Fluvoxamine	1/61 (1.6)	1/11 (9.1)
			Mirtazapine	1/61 (1.6)	1/11 (9.1)
			Trazodone	1/61 (1.6)	1/11 (9.1)
Anxiolytics	3/61 (4.9)	3/11 (15)	Benzodiazepines	3/61 (4.9)	3/11 (27.3)
Stimulants	1/61 (1.6)	1/11 (5)	Methylphenidate	1/61 (1.6)	1/11 (9.1)

**A.** Distribution and cumulative frequency of psychoactive medications used per patient. The table reports the number of patients receiving 1, 2 or more psychoactive drugs, along with the corresponding cumulative frequencies. **B.** Prevalence and distribution of psychoactive medication in the study cohort, including pharmacological classes and specific medications.

The analysis of risk factors for overweight and obesity in univariate and multivariate analysis revealed distinct patterns (**Table 5**). In the univariate analysis for overweight, significantly elevated hazard ratios (HRs) were observed for neuropsychiatric disorders (HR: 4.698; 95% CI: 1.348–16.380) and psychoactive drug consumption (HR: 7.454; 95% CI: 1.602–34.681), while no significant associations were found for sex, heart disease, or thyroiditis. Multivariate analysis, adjusted for sex, confirmed psychoactive drug consumption as the only independent risk factor for the development of overweight (aHR: 7.487; 95% CI: 1.606–34.892). Similarly, in the univariate analysis for obesity, neuropsychiatric disorders (HR: 9.999; 95% CI: 2.611–38.297) and psychoactive drug consumption (HR: 19.25; 95% CI: 3.37–109.97) were strongly associated with increased obesity risk. Interestingly, being overweight was not linked to an increased risk of progressing to obesity (HR: 0.213; 95% CI: 0.061–0.742). The multivariate analysis further identified psychoactive drug consumption as the sole independent risk factor for obesity (aHR: 7.487; 95% CI: 3.532–130.257), while sex did not demonstrate a significant effect.

**Table 5:** Univariate and multivariate analysis of risk factors associated with overweight and obesity.

Univariate analysis for overweight		
Variable	Hazard ratio (HR)	95% CI
Sex	1.086	0.349–3.379
Heart disease	0.362	0.115–1.143
Thyroiditis	1.621	0.453–5.798
Neuropsychiatric disorders	4.698	1.348–16.380
Psychoactive drug use	7.454	1.602–34.681
Multivariate analysis for overweight		
Variable	Hazard ratio adjusted (aHR)	95% CI
Sex	0.884	0.261–2.991
Psychoactive drug use	7.487	1.606–34.892
Univariate analysis for obesity		
Variable	Hazard ratio (HR)	95% CI
Sex	1,68	0,496-5,691
Heart disease	1,056	0,322-3,463
Thyroiditis	0,795	0,189-3,341
Neuropsychiatric disorders	9,999	2,611-38,297
Psychoactive drug use	19,25	3,37-109,97
Overweight	0,213	0,061-0,742
Multivariate analysis for obesity		
Variable	Hazard ratio adjusted (aHR)	95% CI
Sex	2,176	0.497–9,528
Psychoactive drug use	7,487	3,532–130,257

Hazard ratios (HR) and Adjusted Hazard Ration (aHR) and their 95% confidence intervals (CI) from respectively univariate and multivariate analyses for overweight and obesity. Significant risk factors for both conditions include psychoactive drug use, with a notably higher hazard ratio for obesity in both analyses.

**Figure 2:**
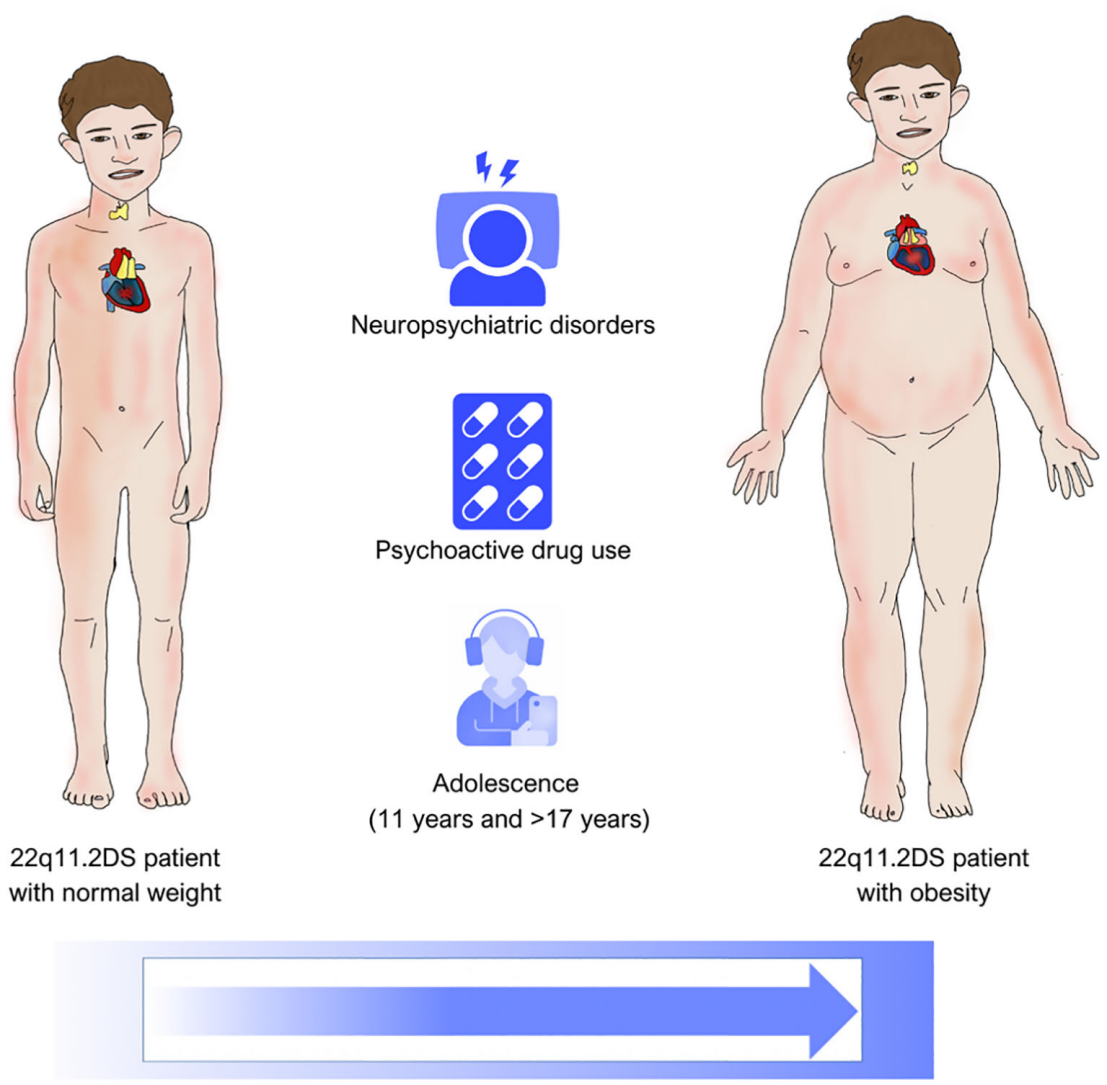
The image depicts the risk of developing obesity in patients with 22q11.2DS. The presence of psychiatric comorbidities, and especially the use of psychoactive drugs, significantly increases the risk, particularly during adolescence, with a bimodal peak at 11 years and after 17 years.

The risk factors for obesity development and the vulnerability ages are illustrated in **Figure 2**.

## Discussion

For years, the literature has debated whether pediatric patients with 22q11.2DS are more likely to become obese than the general pediatric population. However, existing data remain conflicting and are limited by the rarity of the condition and often short follow-up periods.

In one study, the analysis of auxological features in 61 children aged between 4 months and 11 years did not reveal an increased prevalence of overweight (18), whereas another study reported a higher prevalence of obesity among adolescents with 22q11.2DS (21).

Despite the small sample size due to the rarity of the condition, our study supports previous literature findings. Specifically, our data suggest that 22q11.2DS children do not show a greater prevalence of overweight status compared to their healthy counterparts within the entire pediatric population. However, a trend toward a higher prevalence of obesity was observed, with peaks occurring both in late adolescence (age >17 years) and around age 11. This bimodal pattern, with a drop in prevalence at 15 years, could reflect an intrinsic or multicausal susceptibility to obesity in this population.

At the same time, the observed bimodal trend may suggest that the risk of weight gain in patients with 22q11.2DS is not constant but rather characterized by distinct periods of high vulnerability. Such instability could reflect profound psychological adjustment or developmental change, as during puberty, which is a well-recognized period of metabolic and behavioral change (31, 32), potentially amplifying the impact of underlying genetic predispositions or external factors, such as medications. A transient reduction in obesity prevalence at age 15 years could reflect a phase of hormonal stabilization or adjustment in behavior, whereas the rise at age 17 years might coincide with greater autonomy in lifestyle choices and modifications in therapeutic regimens. These findings highlight the need for age-targeted surveillance and interventions to mitigate weight gain during these key developmental phases.

The absence of robust correlations between overweight and obesity and illnesses like thyroid disease, heart disease, and SGA status suggests these biological conditions may not be significant promoters of excess weight gain in patients with 22q11.2DS. Instead, the significant correlation between neuropsychiatric disorders and psychoactive drug use with overweight and obesity highlights the impact of behavioral and pharmacological factors. Neuropsychiatric disorders may affect food habits and exercise, whereas psychoactive medication is already known to induce weight gain. The interaction between psychosocial challenges, functional impairments, or disability, and being overweight has already been well-established in correlation regardless of 22q11.2DS (33, 34).

While neuropsychiatric comorbidities are common in individuals with 22q11.2DS (2, 15, 16), not all patients with psychiatric disorders develop overweight or obesity. Moreover, not all individuals with psychiatric diagnoses are treated with psychoactive medications. However, the initiation of psychoactive drugs markedly increases this risk, as suggested by multivariate analysis, indicating that psychiatric comorbidity alone does not fully account for excessive weight gain. All patients started psychoactive medication before experiencing significant increases in BMI, thus supporting a clear temporal relationship and mitigating concerns about reverse causation or pre-existing obesity.

The contribution of psychoactive drugs to weight and metabolic regulation is well established in the literature (35). It is significant that 58% of the adult patients with obesity included in the Bassett et al. case series had psychiatric conditions (22).

Almost all antipsychotic medications are associated with some degree of weight gain compared with placebo, particularly with long-term use (36–38). Second-generation antipsychotics, such as clozapine and olanzapine, have the highest potential for weight gain, while quetiapine and risperidone are linked to moderate risk and aripiprazole to a lower risk (39).

Mood stabilizers and antidepressants have also been associated with weight gain (40–42). The most dramatic effects on weights were exhibited by mirtazapine and valproate within their categories of drugs respectively, while lithium plays a less significant role (39). Lastly, even anxiolytics such as benzodiazepines indirectly influence weight as they promote a less active and sedentary lifestyle.

Our data underscore the finding that exposure to antipsychotic treatment is the prime risk factor for obesity and being overweight in these patients.

These findings suggest that, unlike other genetic syndromes such as Down syndrome (43), the predisposition to obesity in patients with 22q11.2DS may not be an intrinsic feature but rather a consequence of therapeutic interventions aimed at managing the neuropsychiatric conditions commonly associated with the syndrome.

These results underscore the greatest significance of careful evaluation of the risk-benefit ratio when selecting pharmacologic therapies. Further, it underlines the necessity of careful observation and wise choice of medications for the management of neuropsychiatric symptoms in patients with 22q11.2DS.

While our study provides valuable insights, certain methodological limitations should be considered when interpreting the results. First, the retrospective design did not permit collection of comprehensive lifestyle information, such as dietary habits, physical activity levels, or socioeconomic status, which are well-established contributors to pediatric obesity. These factors could act as confounders, potentially influencing our findings and limiting their interpretability and generalizability. Future prospective studies incorporating these variables could better elucidate the interplay between clinical factors and lifestyle influences on obesity in this population.

Second, our study was conducted in a single tertiary care center, which may introduce selection bias by overrepresenting patients with more complex presentations, including those requiring pharmacological treatment for neuropsychiatric conditions. However, since neuropsychiatric comorbidities are an intrinsic component of the 22q11.2DS phenotype, this potential bias is likely limited. Moreover, the centralization of care for individuals with 22q11.2DS in specialized tertiary centers reflects standard clinical practice, as these patients often require comprehensive diagnostic and therapeutic management. This typical referral pathway reinforces the relevance and generalizability of our findings to the broader 22q11.2DS population.

## Conclusions

In conclusion, obesity risk prevention for 22q11.2DS patients should be proactive, combining early identification of vulnerable time windows and predisposing factors with personalized prevention strategies. Prevention should not be focused only on weight monitoring but also include dietary adjustments, physical activity programs, and psychological support.

Given the complexity of 22q11.2DS patients and their frequent use of psychotropic medications, a multidisciplinary approach—incorporating auxological, nutritional, psychological, and physical care—can significantly reduce the risk of obesity. Moreover, therapeutic choices should prioritize the use of medications with a favorable metabolic profile, balanced by specific interventions aimed at the prevention of excessive weight gain.

In patients under psychoactive therapy, metabolic monitoring should be initiated at the outset in order to detect and treat any potential metabolic derangements. Prevention must be incorporated by pediatricians into an overall management plan, coupled with long-term follow-up in order to provide continuous, preventive maintenance of these patients’ health.

Lastly, caregivers may not always recognize the critical role of weight management, often focusing on more immediate health issues. This serves to underscore the need for urgent education-equipping families with the skills to establish healthy habits from the outset.

## Data Availability

The datasets presented in this article are not readily available because of patient confidentiality and privacy regulations but can be accessed upon reasonable request to the corresponding author, subject to institutional approval. Requests to access the datasets should be directed to matteo.cerutti@unifi.it.
